# Vectorial capacity and vector control: reconsidering sensitivity to parameters for malaria elimination

**DOI:** 10.1093/trstmh/trv113

**Published:** 2016-01-28

**Authors:** Oliver J. Brady, H. Charles J. Godfray, Andrew J. Tatem, Peter W. Gething, Justin M. Cohen, F. Ellis McKenzie, T. Alex Perkins, Robert C. Reiner, Lucy S. Tusting, Marianne E. Sinka, Catherine L. Moyes, Philip A. Eckhoff, Thomas W. Scott, Steven W. Lindsay, Simon I. Hay, David L. Smith

**Affiliations:** a The Wellcome Trust Centre for Human Genetics, University of Oxford, Oxford, UK; b Department of Zoology, University of Oxford, Oxford, UK; c Department of Geography and Environment, University of Southampton, Southampton, UK; d Fogarty International Center, NIH, Bethesda, MD, USA; e Flowminder Foundation, Stockholm, Sweden; f Spatial Ecology and Epidemiology Group, Department of Zoology, Oxford University, Oxford, UK; g Clinton Health Access Initiative, Boston, MA, USA; h Department of Biological Sciences & Eck Institute for Global Health, University of Notre Dame, Notre Dame, IN, USA; i Department of Epidemiology & Biostatistics, Indiana University, Bloomington, IN, USA; j Department of Disease Control, London School of Hygiene and Tropical Medicine, London, UK; k Institute for Disease Modeling, Bellevue, WA, USA; l Department of Entomology and Nematology, University of California, Davis, CA, USA; m School of Biological & Biomedical Sciences, Durham University, Durham, UK; n Institute for Health Metrics and Evaluation, University of Washington, Seattle, WA, USA; o Sanaria Institute for Global Health and Tropical Medicine, Rockville, MD, USA

**Keywords:** Elimination, Malaria, Modelling, Operational research, Policy, Vector control

## Abstract

**Background:**

Major gains have been made in reducing malaria transmission in many parts of the world, principally by scaling-up coverage with long-lasting insecticidal nets and indoor residual spraying. Historically, choice of vector control intervention has been largely guided by a parameter sensitivity analysis of George Macdonald's theory of vectorial capacity that suggested prioritizing methods that kill adult mosquitoes. While this advice has been highly successful for transmission suppression, there is a need to revisit these arguments as policymakers in certain areas consider which combinations of interventions are required to eliminate malaria.

**Methods and Results:**

Using analytical solutions to updated equations for vectorial capacity we build on previous work to show that, while adult killing methods can be highly effective under many circumstances, other vector control methods are frequently required to fill effective coverage gaps. These can arise due to pre-existing or developing mosquito physiological and behavioral refractoriness but also due to additive changes in the relative importance of different vector species for transmission. Furthermore, the optimal combination of interventions will depend on the operational constraints and costs associated with reaching high coverage levels with each intervention.

**Conclusions:**

Reaching specific policy goals, such as elimination, in defined contexts requires increasingly non-generic advice from modelling. Our results emphasize the importance of measuring baseline epidemiology, intervention coverage, vector ecology and program operational constraints in predicting expected outcomes with different combinations of interventions.

## Introduction

Billions of dollars are spent on vector control each year to reduce transmission of malaria and other mosquito-borne pathogens.^1^ Despite huge investments, questions remain about the likely effects of scaling-up various modes of vector control and the optimal mix of interventions, particularly when the final push for elimination is made.^2,3^ Here, we review the various theoretical updates to vectorial capacity (VC) and critically evaluate the mathematical basis of the quantitative concepts most commonly used to inform vector control policy.

Vector control followed logically from discoveries in the decades around 1900 that mosquitoes transmit filariasis, malaria, yellow fever, dengue and other pathogens.^4^ During that period, larval source management (LSM) was commonly undertaken as a way of controlling transmission of malaria, yellow fever and dengue,^5^ and bednets and screens—interventions already in use to reduce nuisance mosquito biting—were repurposed for disease control.^5,6^ Ronald Ross meanwhile developed a mathematical model of LSM,^7^ and two models (unrelated to the LSM model) describing malaria transmission.^8,9^ In the 1940s, with the invention of indoor residual spraying using DDT, new methods for vector control were developed.

**Figure 1.**
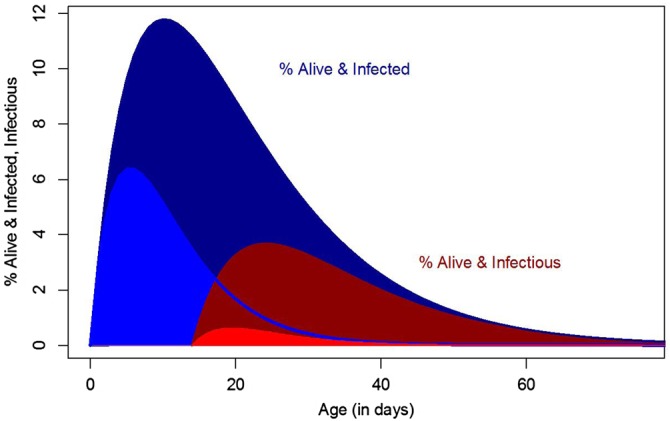
Simulated output from Macdonald's model of sporozoite rates.^6,10^ Curves show the percent of a mosquito cohort that is alive and infected (blue) or infectious (red) for a baseline (darker shades) and with doubled mortality rates (lighter shades). The area under the red curves is proportional to total transmission per adult mosquito. These curves assume approximately 10% of mosquitoes become infectious after biting a human, and *f=(3 days)*^−1^; *Q=95%*; *g=1/12 days*^−1^; *n=14 days*. Changes in the area under the curves are well described by a simple elasticity analysis.

All of these developments set the stage for George Macdonald's impactful synthesis of medical entomology,^10^ including a mathematical model for the sporozoite rate^10^ (Figure 1), definition of the basic reproductive number for malaria (*R*_0_),^6,10–12^ and explanation of the relevance of these concepts for malaria eradication.^13^ Macdonald's analysis identified those elements of a mosquito's life history that matter most for his transmission model through a concept that came to be known as the ‘daily reproductive number’ or VC.^12,14,15^ Vectorial capacity was defined by a formula (Box 1) describing the total number of potentially infectious bites that would eventually arise from all the mosquitoes biting a single perfectly infectious (i.e., all mosquito bites result in infection) human on a single day.^15^ The vast majority of mathematical models describing pathogen transmission by mosquitoes make similar assumptions to Macdonald's model.^14^ Consequently, understanding VC is equally relevant today as it was half a century ago.

Box 1. **Classical vectorial capacity**

Four parameters comprise the classical formula for vectorial capacity (*V*): the parasite's extrinsic incubation period (EIP, *n* days); the ratio of mosquitoes to humans (*m*); mosquito survival through one day (*p*); and human biting rates (*a*):

(B1) $$V=\frac{ma^{2}\;p^{n}}{-\ln\;(p)}.$$

An intuitive restatement of the Eqn. (B1) is that an infectious person will be subject to the attention of *m* mosquitoes (assuming everyone is equally attractive) and will receive *ma* bites each day. For those mosquitoes to become infectious they must survive the extrinsic incubation period (with probability *p^n^*). The adult mosquitoes (on average) live for 1/(−ln(p)) days biting, and potentially infecting, humans at a rate of *a* per day. Eqn. (B1) combines these quantities to give the total potential infectious bites arising from one infected person for one day.

A comparison between Eqn. B1 and Macdonald's original derivation reveals a mathematical inconsistency.^16^ Macdonald's model was formulated in continuous time with constant mosquito death rates, here denoted *g*, with expected lifespan (1/*g*). Had the model been formulated using a daily time step, the average mosquito lifespan would be $(1-p{)}^{-1}.$ Macdonald simply re-parameterized^6^: $p=e^{-g}$, and g=−ln(p).

Macdonald's analysis of the formula for VC showed that malaria transmission should be highly sensitive to adult mosquito survival,^10^ and it helped explain reasons for the success of early DDT spraying programs in the late 1940s and 1950s in terms of a sensitivity analysis. He argued that DDT reduced survival of adult mosquitoes, and survival affected transmission by both reducing the number of infectious bites and reducing the number of mosquitoes that survive the parasite's extrinsic incubation period.^11,16^ This analysis helped explain why indoor spraying with DDT had worked so well in early field trials and justified expansion of indoor residual spraying (IRS) programs to attempt malaria eradication in the 1950s and 1960s before it was known if these goals were technically, operationally or financially achievable.^13,16,17^ Despite the end of the Global Malaria Eradication Program, Macdonald's simple sensitivity analysis has had a long, profound, and ongoing influence on malaria control policy.^18,19^ Consistent with Macdonald's analysis, the standard advice has been to adopt interventions that shorten the lifespan of adult mosquitoes; hence, insecticide treated bednets (ITNs) and IRS are usually recommended over LSM^19^ or other interventions targeted at immature stages.

The conclusions reached from Macdonald's model are by no means analytically incorrect. Instead, what we must now question is how well this simple model represents the realities of the increasingly diverse and complex malaria transmission environments in which contemporary control policy decisions must be made. These situations require a consideration of: practicalities of operational constraints (administrative and logistical constraints on delivering and maintaining effective interventions to target populations^2^); the challenges increasingly posed by insecticide resistance and residual transmission^20,21^; and the complications of achieving high coverage with vector-based interventions. All of these must be considered in transmission settings with different baseline epidemiological and entomological characteristics and different target effect sizes. Given this, if we modify models in appropriate ways to more accurately represent these setting-specific characteristics, do our conclusions change regarding the optimal mix of interventions?

With an ever-increasing focus on explicitly setting and evaluating control program outcomes, the role of the concepts of VC and *R*_0_ are becoming increasingly important tools to understand the roles of different interventions. This is particularly important in elimination settings, where reducing $R_{0}\;<1$ is a threshold condition for cessation of local transmission.^22^ With these quantitative endpoints there is a need to re-assess whether simply scaling-up preferred interventions will be sufficient to reach this goal, or whether the challenges posed by each different setting will require carefully considered strategic adjustments.

## Methods and Results

### What classical theory tells us about control

A contemporary update of Macdonald's sensitivity analysis evaluates proportional reductions in transmission, called effect sizes $\left(E_{C}\right)$, defined by the ratio of baseline VC ($V_{0}$) to its value with some vector control ($V_{C}$), so $E_{C}=V_{0}/V_{C}$.

The formula for VC has also evolved and it is rewritten in previous work to separate the effects of larval and pupal mosquito ecology in aquatic habitats from those of flying, adult mosquitoes, and to consider population dynamic feedbacks (Box 2).^12,23–25^ Macdonald's argument about why adult mortality was so important can also be updated through elasticity analysis (Box 3), which describes the sensitivity of effect sizes to proportional changes in the parameters comprising VC (Table 1).

**Table 1.** Summary of the mathematical order of parameters and terms using various formulae for vectorial capacity (See Box 1 and Box 2)

	Vectorial Capacity, *V*	$\epsilon_{V}(m_{0})$ (Introduction of a refractory gene in the adult mosquito population)	$\epsilon_{V}(\lambda_{0})$ (Larval source management)	$\epsilon_{V}(G_{0})$ (Delayed blood feeding or increased adult mortality)	$\epsilon_{V}(n_{0})$ (Bacterial symbiont that delays parasite maturation)	$\epsilon_{V}(Q_{0})$ (Non-insecticide treated bednets)	$\epsilon_{V}(\;f_{0})$ (Barrier methods targeted to all hosts)	$\epsilon_{V}(g_{0})$ (Insecticide-based methods)
Ross-Macdonald^11^	$\frac{ma^{2}p^{n}}{-\ln(p)}$	1	0	0	*gn*	2	2	1+*gn*
Smith and McKenzie^6^	$\lambda \frac{\;f^{2}Q^{2}e^{-gn}}{g^{2}}$ or $\lambda S^{2}P$	1	1	0	*gn*	2	2	2+*gn*
Current analysis	$\lambda (G)S^{2}P$	1	1	*o*	*gn*	2	2+*o*	2+*o*+*g*

Example interventions are given for each parameter. Where local vector populations are robust and have relatively little immigration o≈1 and will increase or decrease depending on the relative importance of internal or external dynamics for population persistence. The elasticity of *gn* depends on the ratio between extrinsic incubation period (EIP) *n* and mosquito lifespan 1/*g*. If the two are approximately equal, then gn≈1. If EIP were half of mosquito lifespan (i.e.gn≈0.5), then the elasticity would scale as a square root, and were it twice as long (*i.e.*gn≈2), elasticity would be quadratic, of order 2.

Box 2. **Updating vectorial capacity**

The formula for vectorial capacity has been extended in two ways.^6,25^ When it was first described as a separate quantity, human biting was decomposed into overall biting rates (*f*), and the proportion of bloodmeals on humans (Q),^15,26^ such that a=fQ. Later, the ratio of mosquitoes was eliminated by introducing a parameter, λ, that describes the number of adult mosquitoes that emerge from larval habitats, per human, per day,^25^ such that dm/dt=λ−gm, and the steady state would be m=λ/g. Vectorial capacity can be written in a way that separates the effects of larval and pupal mosquito ecology in aquatic habitats from those of adult mosquitos:

(B2.1) $$V=\lambda \frac{\;f^{2}Q^{2}}{g^{2}}e^{-gn}.$$

Interpreting this formula is made simpler by combining parameters describing adult behavior into two terms: the expected number of human blood meals a mosquito would take in its lifetime (S=fQ/g); and the probability a mosquito survives through the EIP ($P=\;p^{n}=e^{-gn}$):

(B2.2) $$V=\lambda S^{2}P.$$

In B2.2, the term describing lifetime biting appears twice: after emerging, one blood meal infects the mosquito, and after surviving the EIP, another infects a human.

Further extensions of the basic model consider feedbacks between adult female blood feeding and egg laying and larval ecology. Let *v* denote the number of eggs laid per female, the expected number of eggs laid by a mosquito over its lifetime is G=νf/g, so a comprehensive analysis of adult vector control must consider:

(B2.3) $$V=\lambda (G)S^{2}P.$$

The order of this effect depends on ecology, including population dynamic thresholds and mosquito migration.^22^

Box 3. **Elasticity**

The effect size associated with changes in a parameter *x* is defined by its baseline *x*_0_ and its new value under control *x*_c_:

$$E_{V}(x_{C}|x_{0})=\frac{V(x_{0})}{V(x_{C})}.$$

The effect sizes associated with large changes in *x* can be evaluated directly using the formulas, but some useful insights come from a sensitivity analysis, which looks at the changes in *E* associated with small changes in *x* around the baseline:

$${\left.\frac{dE_{V}(x|x_{0})}{dx}\right|}_{x=x_{0}}=-\frac{V^{\prime }(x_{0})}{V(x_{0})}.$$

Since an effect size is defined as a proportional change in transmission, it is of greater interest to look at the elasticity, the sensitivity to small proportional changes in *x* around baseline, which is defined by the following:

$${\left.\epsilon_{V}(x_{0})=\frac{dE_{V}(\theta x_{0}|x_{0})}{d\theta }\right|}_{\theta =1}=-x_{0}\frac{V^{\prime }(x_{0})}{V(x_{0})}.$$

Three rules make it trivial to compute the elasticities of the parameters and functions in any formula for vectorial capacity that does not explicitly consider the effects of mosquito population dynamics:

1. If $V(x)=bx^{k}$, where *b* is any constant, then ϵ(x)=−k.
2. If $V(x)=be^{-xy}$, then $\epsilon_{E}(x)=xy,$so the elasticity of *x* depends on *y*.
3. Elasticities are additive, for if V(x)=f(x)g(x), then $\epsilon_{V}(x_{0})=$ $\epsilon_{f}(x_{0})+\epsilon_{g}(x_{0})$.

As shown by previous work,^23^ changes in VC are linearly proportional to changes in mosquito population density: such effects are called 1st order (Table 1). This can be seen from inspection of the formula for VC because the parameter *m* appears by itself (*i.e.m^1^*) and only once (Box 1). Interventions that reduce adult mosquito density, such as environmental management of larval sites would have 1st order effects. Genetically modifying mosquitoes to render them refractory to infection would also only have a 1st order effect assuming refractoriness is complete, the frequency of refractoriness remains stable and mosquito immature development is unaffected by moderate changes in abundance. It follows that, halving mosquito population density or the proportion susceptible to infection from humans would halve VC (i.e., to 50% of baseline, or an effect size of 2).

In comparison, the parameter for the human blood index (proportion of bloodmeals taken on humans, HBI) (Q) appears twice ($Q^{2}$) in the updated VC equations (Box 2), so they have a 2nd order effect. Diverting half of blood meals onto non-human hosts through, for example, repellents or zooprophylaxis, would have an effect size of 2^2^=4 (i.e., a 75% reduction).

The mosquito biting rate (*f*) appears twice in the revised equation (*f*^2^) (Box 2), but reductions in this rate have the additional effect of reducing the number of eggs laid, and after considering density-dependent effects in immature mosquito habitats, the number of adults emerging and mosquito population density. In further developments of these equations^23^ (Table 1), the order of this effect is denoted by *o*, and the overall effect of reducing biting rate is 2+*o*. In systems with a common power-law mortality response to increased density, egg laying has a 1st order effect on mosquito population density (*i.e.* o≈1).^23^ This means that reducing feeding rates (*f*) would have a 3rd order effect on VC. Doubling the interval between blood meals through, for example repellents targeted to all host species, would have an effect size of 2^3^=8 (i.e. a 87.5% reduction).

Mosquito lifespan affects the number of eggs laid and the probability of surviving the extrinsic incubation period (Table 1). In Macdonald's formula (Box 1), changes in mosquito lifespan have order 1+*ng*, but his analysis missed two additional effects of increasing mosquito mortality that are apparent after expanding the formula (Box 2, Table 1): reduced adult density through shorter life-spans and reduced egg laying leads to fewer adults in the next generation. This means the overall effect is of order: 2+*ng*+*o*. Changes in mosquito lifespan, where ng≈o≈1, have a 4th order effect.^23^ Halving mosquito lifespan would have an effect size of more than 2^4^=16 (i.e. >95% reduction, Figure 1). Relaxing the assumption of constant adult mortality to include senescence would also further increase this effect size.^6,26,27^

The robust general conclusion from elasticity analysis is that VC is most sensitive to proportional changes in mosquito mortality (of order 2+*ng*+*o*), followed by changes in overall feeding rates (of order 2+*o*), human feeding habits (of order 2), and last of all mosquito population density (of order 1) (Table 1).

### Understanding control in specific cases: reduced effect sizes in complicated systems

Analysis of elasticity and effect sizes offer a sharp, elegant mathematical insight, but the question addressed by this specific manuscript is whether these robust conclusions arising from mathematical analysis are relevant for making public health policy. Policy analysis considers questions related to the distribution of interventions and health outcomes, not sensitivity to parameters. Whether focused on transmission suppression (control) or elimination, success will depend on the type and coverage of existing interventions, the capacity for these interventions to be scaled up and the potential to introduce new interventions.

Until recently there were few attempts to explicitly model effect sizes in response to intervention coverage levels, but the modern concept of effect size has been updated in recent publications describing effect sizes of ITNs,^28–32^ IRS^32–34^ and LSM.^23,33^ Models describe the effect sizes in relation to effective coverage levels, ϕ, which are broadly defined as the proportion of the relevant events or quantities in a mosquito population affected by the intervention.^35^ A variety of detailed definitions of effective coverage are currently in use that take into account coverage beyond human blood sources,^36^ heterogeneity in human-mosquito contact^37^ and community-wide protective effects^38,39^ that expand upon what are termed here as epidemiologically relevant events or quantities. By taking these events into account effective coverage levels can be quantified and, in the majority of settings, distinguished from intervention/demographic/household coverage levels (e.g., ITN ownership multiplied by usage).

Reformulating models of effect sizes in relationship to intervention coverage levels and effective coverage levels, $E_{\phi }$, highlights the importance of the relationship between technical, operational and ecological aspects of malaria control programs. Policy options will typically be compared on the basis of which option achieves the required effect size for the least effort. The generic term ‘effort’ is used here in reference to technical challenges of deploying operational resources to the target population and is used as a simpler alternative to ‘cost’. Consideration of intervention costs is itself a further development given the additional non-linear factors that need to be taken into account such as economies of scale and time discounting.

**Figure 2.**
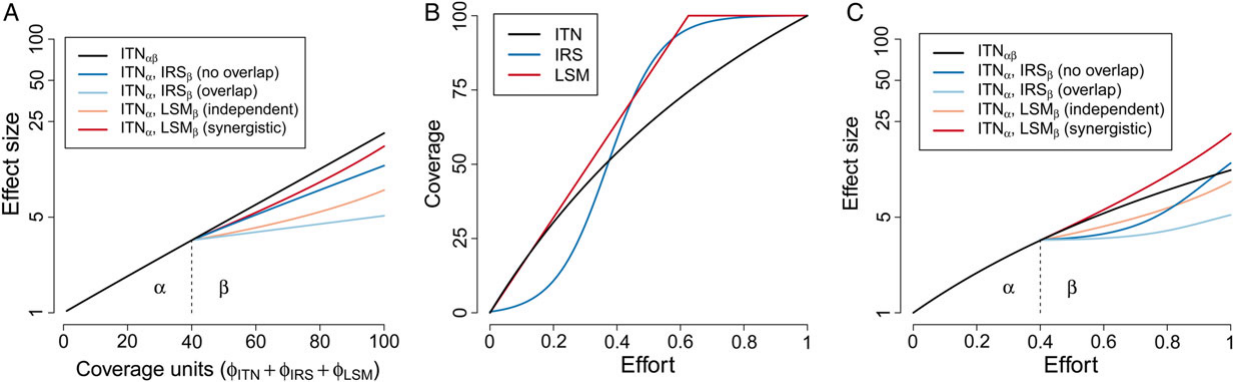
Changing choices when the technical challenges of achieving coverage levels with different interventions are taken into account. Most models consider how increasing coverage (ϕ) will alter effect size (A), but the effort needed to achieve a given increase in coverage may vary depending on intervention and baseline coverage (B). This may mean that if control program decisions are budgeted by effort (e.g., economic costs or the time commitments of skilled personnel) instead of coverage, the optimal choice of interventions may change (2C). The above considers an initial phase (α) where insecticide treated bednets (ITNs) are scaled up to 40% coverage. In a more intensive second phase, (β), either an additional 60% of the population will be covered (A), or one and a half times the effort expended to reach the 40% coverage with ITNs will be invested (2C). In each of these scenarios the following intervention combinations are available: switch to IRS which has a similar, but slightly less effective, mode of action to ITNs, which, depending on the logistics of deployment, may reach completely different (no overlap in Figure 2) or half overlap (overlap in Figure 2) with those who are already covered by ITNs; switch to larval source management (LSM) which has a different mode of action to ITNs and, depending on mosquito population dynamics, may have independent or synergistic effects in combination with ITNs; continue scaling up ITNs.

Consider scale-up of a control program in two phases (Figure 2), where in the first phase (α) an intervention such as ITNs is scaled up to 40% coverage. In the second phase (β), a program could switch to scaling up coverage of different interventions (IRS or LSM), or take steps to further increase coverage of ITNs, likely with increasing marginal effort. In these models, the critical consideration is how the parameters in vectorial capacity are affected by the presence of both interventions at their respective coverage levels. Quantifying how long mosquitoes survive and how often they feed when there are two or more interventions present measures the gap between intervention coverage and effective coverage.

The expected effect size of alternative interventions will depend on their interaction with the interventions already in place in terms of both the parts of the transmission cycle they affect and whether these new interventions cover different individuals. If an intervention with a similar, but slightly less effective, mode of action were chosen (e.g., IRS), the effect size will be highly dependent on it being deployed to those missed by ITNs (no overlap vs overlap in Figure 2A). If an intervention has a different mode of action (e.g., LSM in the presence or absence of strong larval density dependence), effect size will principally depend on whether it acts independently from or synergistically with ITNs (independent and synergistic Figure 2A). In many cases, the coverage gaps being addressed may be caused by the behaviors of minor vector species, such as outdoor feeding (see below).

While switching interventions may lead to lower initial effect sizes (Figure 2A),^40^ concentrating only on coverage may be misleading as achieving high coverage may come at very different effort investments for different interventions (Figure 2B). Many models assume a simple linear relationship between effort and coverage, e.g., LSM has a fixed cost per unit area and mosquitoes are distributed homogeneously. Many interventions, however, have high initial set up costs, but increasing coverage becomes cheaper the more effort is invested, e.g., equipment or personnel investments to conduct IRS. Finally, and perhaps more commonly with a number of interventions, effort needs to be increased to maintain the same increases in coverage, e.g., delivery of ITNs to inaccessible communities (Figure 2B). The specific dynamics of the cost to coverage relationship for each intervention will be highly specific to local epidemiology and how existing distribution mechanisms can be utilized. Control programs are budgeted by cost and operational resource constraints, not coverage. If the choices of interventions are reassessed with a fixed effort budget instead of fixed coverage budget, the effect size achievable with different options changes (contrast Figures 2A and 2C).

In reality this decision may be made even more complex as combinations of interventions may transition from overlapping to synergistic depending on the coverage of each intervention (the changeable gap between coverage and effective coverage). This means that while the lines shown in Figure 2 may show the upper and lower bounds in effect size of an intervention combination, the actual effect size will depend on a highly non-uniform 3D surface with coverage of the two (or more) interventions on *x* and *y* axes and effect size on the *z* axis. Characterizing these surfaces is important for understanding how integrated vector control would work in different settings.

**Figure 3.**
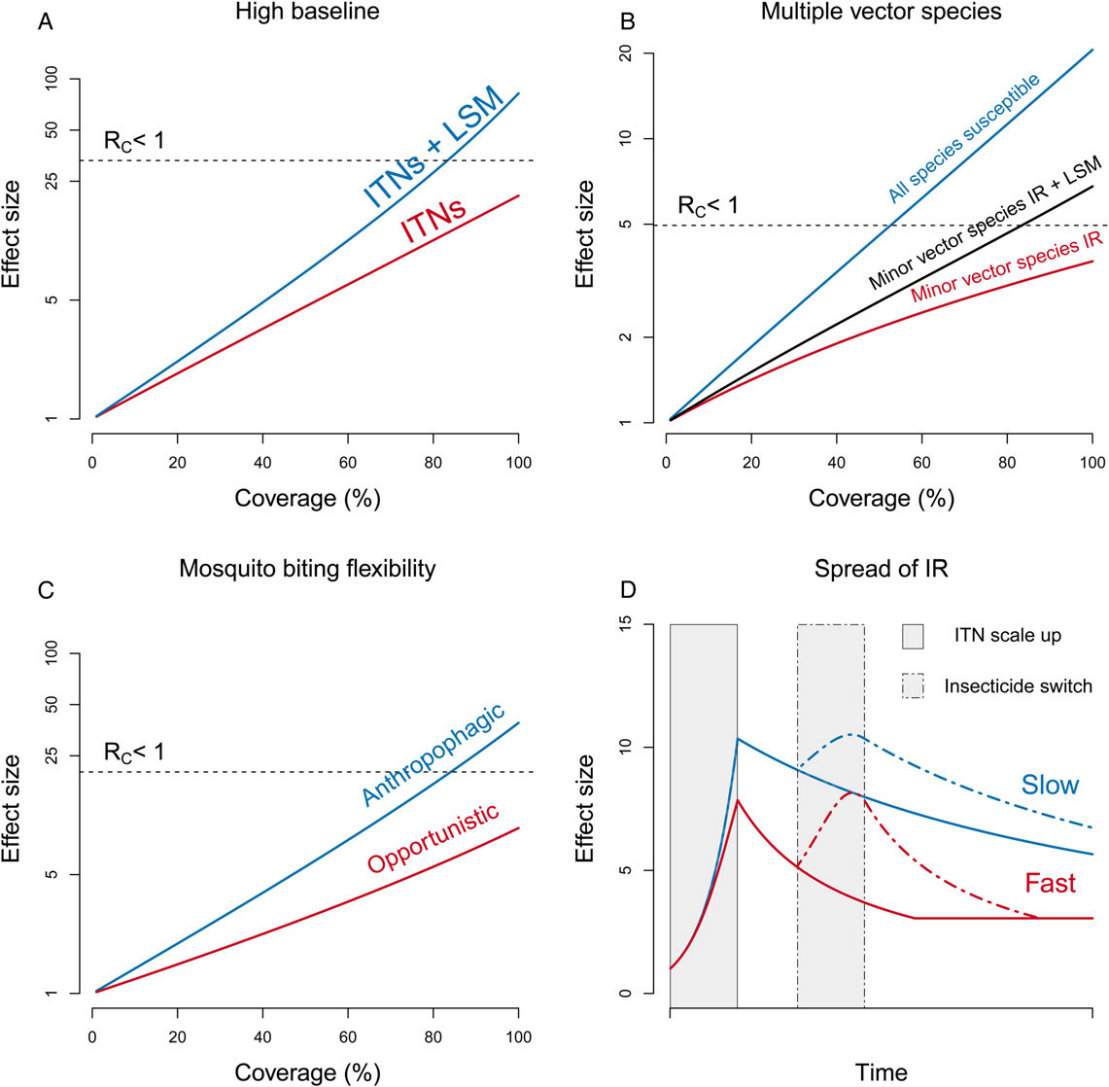
Challenges of meeting policy goals in different epidemiological contexts. Policy goals generally involve reducing transmission down to some target level. In the case of elimination, this requires reaching an effect size sufficient to reduce R_C_<1 (i.e., above the dotted line in A–C). Under certain situations this cannot be achieved through scaling-up coverage of a single intervention alone, including: (3A) high baseline transmission (insecticide treated bednets [ITNs] and larval source management [LSM]); (3B) multiple vector species (red and black lines denote a setting where half of vectorial capacity (VC) is due to a species that is insecticide resistant [IR] but still susceptible to LSM in comparison to the blue line where all species are susceptible to all interventions); (3C) mosquito biting plasticity reduces the effectiveness of ITNs (in the red line feeding frequency is unaffected in mosquitoes with opportunistic biting patterns due to the availability of non-human hosts); (3D) the spread of insecticide resistance (plots show the change in effect size as ITN coverage is scaled up to 80% [grey shaded bar] then resistance emerges at half the rate of ITN scale up [fast, red line] or one tenth the rate of scale up [slow, blue line]). Dotted lines show the effect of a second ITN campaign where nets are replaced with a different insecticide.

In addition to operational constraints, particular features of the biological systems concerned may complicate reaching a particular effect size. In settings with high baseline transmission, achieving a particular goal may not be possible with a single intervention, irrespective of how effective it is, and other methods must be called on to fill in the effective coverage gaps (e.g., adding LSM to ITNs, Figure 3A).

Reaching some target effect size in areas that include vectors refractory to control is also a particular concern.^39^ Vector refractoriness to control can include physiological resistance (i.e., insecticide resistance) or behavioral resistance (e.g., outdoor resting and biting plasticity) that minimize the effectiveness or contract with interventions, respectively.^20,21,41^ A further distinction can be made to distinguish resilient vector populations, that have pre-existing traits that make them refractory to control, from those that develop resistance in response to control efforts.^42,43^ Such systems are common, perhaps even a majority, in elimination settings,^44–48^ and the refractoriness leaves a gap to be filled. This gap can be illustrated with simple mathematics.

Where there are multiple vector species or types, total VC is the *sum* of the VCs of individual species or types:

Since the total effect size is found only after adding up vectorial capacities, effect sizes are no longer multiplicative when vectors respond differently to control. Arguments based solely on mathematical sensitivity or elasticity of VC would, therefore, again not tell the whole story.

To illustrate this, consider two vector populations (*S* and *r*) that respond very differently to some intervention. If one of those species (say *S*) is highly sensitive (such that $V_{\phi ,s}=0$), and the other is completely refractory (i.e. $V_{\phi ,r}=V_{0,r}$), then the total effect size is:

$$E_{\phi }=\frac{V_{0,s}+V_{0,r}}{V_{\phi ,s}+V_{\phi ,r}}=1+\frac{V_{0,s}}{V_{0,r}}$$

In this example, effect size of the intervention is determined by the more refractory species, and the ratio of VCs sets a maximum effect size. If this maximum effect size falls below the target effect size, other intervention methods may be required to target the refractory species (e.g., using LSM to target pyrethroid-resistant species, Figure 3B). Such situations have already been extensively observed by monitoring long-term trends in species-specific abundance and intervention coverage.^39,43,49^ One, by no means exclusive, example was the significant decline in *Anopheles gambiae* s.s., but relatively unaffected *An. arabiensis* populations in southern Kenya following moderate ITN scale up.^50^ This difference in refractoriness has even been extensively observed within species with, for example, different populations of *An. farauti* exhibiting different preferences for indoor and outdoor feeding.^51^

A similar pattern is observed when species exhibit behavioral resistance that diminishes or removes a method of action of a particular intervention. While there is some debate over whether observed behavioral resistance reflects genuine changes in a single species' behavior or increasing relative abundance of other species with differing behaviors,^42^ the effect on overall VC is similar and a shift towards more blood meals coming from non-human hosts (opportunistic, Figure 3C) can substantially limit the maximum effect size that is achievable with a single intervention (Figure 3C). In such cases, controlling residual transmission will require adding an intervention that targets refractory behaviors attributable to specific vector species.

As a final example, the spread of insecticide resistance can affect the maximum effect size that can be reached and the timescale over which interventions need to be scaled up. If an insecticide resistance phenotype was related to the frequency of a gene that was evolving over time (i.e., *q_t_*), and if selection were a function of effective coverage and other uses of the insecticide, $q_{t+1}=F(q_{t},\phi_{t},...)$, then the gaps would grow as insecticide resistance evolved according to the equations:

$$E_{t,\phi_{t}}=\frac{(1-q_{t})V_{0,s}+q_{t}V_{0,r}}{(1-q_{t})V_{\phi ,s}+q_{t}V_{\phi ,r}}$$

The sustainability of a particular effect will depend on the ratio between the rate of effective coverage scale up and the rate of insecticide resistance spread. Depending on the fitness penalties of control and resistance, the rate of insecticide resistance spread may be dependent on the rate of effective coverage scale up due to selection intensity. Figure 3D shows two simulations where an ITN campaign is scaled up to 80% effective coverage over time with resistance to the insecticide developing at half the rate of intervention scale up (red line, fast scenario) and one tenth the rate of intervention scale up (blue line, slow scenario). The two peaks in effect size (blue and red lines in Figure 3D) followed by declines at different rates show how the rate of insecticide resistance spread can have an effect on both the maximum effect size that can initially be reached and the total effectiveness over time. The current spread of pyrethroid resistance in southern Africa and the observed decline in ITN effectiveness and effective lifetime has demonstrated the importance of monitoring insecticide resistance and its impact on the effectiveness of specific interventions.^52,53^ When insecticide resistance limits achievable effect size, the best option may be to switch insecticides, even if it results in lower effective coverage for similar costs.

Replacing the active component of a particular intervention, such as switching insecticides used for IRS (dashed lines, Figure 3D), can be used to prolong the duration of effectiveness. In this case the required frequency of insecticide rotation will depend on both the minimum effect size that must be obtained at all times (the troughs in effect size) and the rate at which insecticide resistance develops. Quantifying the fitness costs of a particular resistance trait will be key in optimizing rotation frequency and the number of different insecticides required in relation to optimal effective coverage. Finally, while difficult to measure, it is also important to consider that other environmental complexities, such as intensive use of agricultural pesticides or general pollutants in water bodies, may have a potential effect on baseline resistance profiles. In such cases, reaching a desired effect size will require a wider consideration of ecological management.

In the above increasingly common cases, it is important to reiterate that methods that maximize adult mosquito killing are still likely to be an important part (if not the most important part) of an effective control campaign. What these considerations do suggest, however, is that when making the decision to further scale up vector control, total effective coverage, costs, and effectiveness of all possible options need to be considered in the present and evolving context rather than simply relying on what has worked in the past.

### Setting achievable goals: interpreting effect sizes and policy outcomes

Vectorial capacity describes how local vector populations determine the intensity of transmission, while effect sizes describe how they change it. Achieving some policy objective through vector control generally involves reducing VC from its baseline down to some lower level then sustaining those effects for some time.^54–57^ These policy objectives are generally interpreted through the use of mathematical models, and the relevance of VC is interpreted through its effective reproductive number (*R_C_*). *R_C_* establishes a threshold condition for pathogen persistence in mathematical models, and these thresholds provide a basis for setting target effect sizes in relation to policy objectives. In a policy context, effect sizes are only relevant in relation to the baseline which must consider transmission in the absence of any control (i.e. *R_o_* ), or in the presence of some interventions (*i.e. R_C_*_1_) for an accelerated campaign.^10,11,31,58^ Assuming the human-mosquito (*b*) and mosquito-human (*c*) transmission probabilities remain unaffected by control, the effect size difference between VC and *R_C_* is solely dependent on how other interventions, such as treatment with anti-malaria drugs, affect the duration of human infectiousness *D*:

$$E_{C2}=\frac{R_{C1}}{R_{C2}}=\frac{V_{C1}D_{C1}}{V_{C2}D_{C2}}\;$$

To reach a predetermined endpoint, transmission must be further reduced down to some level (*R_C_*_2_), with the ratio $R_{C1}/R_{C2}$ giving the target effect size. While the success of malaria elimination-specific policies may be evaluated based on *R_C_*,^59^ control programs are more frequently evaluated through changes in prevalence, clinical incidence or deaths.^60^ Extending the concepts developed here to these increasingly variable measures would require a detailed consideration of human immunology, treatment seeking behavior and clinical management,^61,62^ and of the way these metrics scale.^63,64^

### Troubleshooting control programs with unexpected outcomes

Mass distribution of interventions has reduced transmission in many places, but despite high intervention coverage, prevalence of malaria infection has remained high or higher than expected in some areas.^53,65,66^ By combining medical intelligence with the principles and notions of effect sizes, policymakers in these situations can reanalyze existing policy to understand why these interventions had an unexpected effect and to revise expected policies and goals (Box 4).

Box 4. **Measuring baseline and re-evaluating effectiveness**

Measure at baseline:

- Malaria transmission intensity
  - EIR
  - *R*_0_
  - Infectious reservoir and proportion asymptomatic
  - Prevalence
  - Clinical incidence
- Local vector species
  - Relative abundance
  - Differing bionomics
- Vector behavioral resistance traits
  - Vector time allocation (host-seeking vs non-host seeking)
  - Outdoor/daytime biting and outdoor resting
- Vector physiological resistance traits
  - Insecticide susceptibility to each insecticide class
- Technical operational constraints
  - Peak coverage
  - Rate of scale up
  - Effectiveness decay over time and rate of replacement

Measure at intervals to re-evaluate expected effect size and strategy effectiveness:

- Malaria transmission intensity
  - EIR under control
  - *Rc*
  - Infectious reservoir and proportion asymptomatic
  - Prevalence
  - Clinical incidence
  - Malaria importation rate
- Coverage levels
  - Expected *vs.* actual population coverage
  - Coverage evenness
  - Effectiveness of interventions among those covered
- Vector behavioral resistance traits
  - Changes in biting preferences (through repeated testing)
- Vector physiological resistance traits
  - Rate of spread of resistance (through repeated testing)
  - Prevalence of key mechanisms of resistance

In weighing a response, the first question that should be asked is what intervention effective coverage was actually achieved and what effect size was actually obtained? The failure to reach a policy goal might be the result of lower than expected effective coverage (e.g., operational programmatic failures or low usage of interventions) or effectiveness (e.g.*,* insecticide resistance or mosquito behavioral plasticity) and each of these could have changed over time. Such phenomena can be measured using a range of field assay techniques.^67,68^

In measuring an effect size some consideration should also be given to the way the outcome was measured. Most measures of human infection or disease respond in a highly non-linear way to changes in VC. This means that that a 10-fold reduction in baseline VC would translate into much smaller changes in the incidence of human infection, clinical malaria or prevalence.^69^

Knowing why a single intervention failed would tend to inform the decision of what to do next. If an ITN distribution failed but there was a high frequency of insecticide resistance, for example, then the next step might be to change the insecticide in the nets.^63,70^ In areas where transmission intensity is particularly high, it may be necessary to add interventions or improve coverage with existing interventions to reach the desired goal. Updated models that include this new baseline can then be used to support adapted control program strategies and targets.

## Discussion

By analyzing the formulas for VC in various real world malaria transmission settings we have shown that there are many situations where concentrating exclusively on methods that kill adult mosquitoes may not be the best way to reach a desired policy endpoint. Macdonald's classical parametric sensitivity analysis remains useful as a principle for identifying which methods are likely to have the biggest effect on transmission. Translating advice from malaria transmission models into policy action requires an understanding of the relationship between intervention coverage levels, VC, *R_C_* and measures beyond including how this might vary by context. The relationship between effect size and required intervention coverage targets can be complicated in many different ways through changeable mosquito bionomics and the operational constraints of different interventions. What follows from such an approach is that the overall strategic planning for attacking mosquito-borne pathogens should give greater consideration to fully characterizing the baseline epidemiological and entomological characteristics of a given setting (Box 4). Identifying likely effective coverage gaps, and considering the interplay between the technical requirements and operational capacity are also crucial.^71^ As a result, policy must take into account the baseline as a factor affecting the optimal choice of interventions and the outcome that can be reached.^40,58^

A more complete consideration of the models with their adaptations to different transmission settings exposes well-supported reasons to avoid universal application of Macdonald's original analysis and its over-reliance on the concept of mathematical sensitivity to parameters under a single, overly simplistic model. Recent analysis has explicitly considered mosquito population dynamics and LSM.^23^ Though the models generally concur that the emergence rate of adult mosquitoes has a linear effect on mosquito density, they also suggest that reductions in mosquito density could respond in a highly non-linear way when intervention coverage is increased.^25^ Mosquito population regulation at different life-cycle stages remains a major gap in our understanding of vector ecology despite its core relevance for modelling how many interventions work. In addition we should acknowledge the limitations in understanding that can be gained from modelling alone, particularly in its ability to give conclusive answers in a local context. Modelling and malaria control theory will never be a replacement for consistent, reliable and ubiquitous field data collection. The prospects for success of a given control program will often depend on particular features of mosquito and pathogen ecology, which must be measured at baseline (Box 4).^39^

In any case, the proper basis for comparing vector-based interventions is not the mathematical order per se, but the effect size that would come from reaching coverage levels with different interventions at comparable costs. For example, available evidence suggests that LSM achieves comparable reductions in transmission for comparable costs to LLINs and IRS.^72^ A more general consideration of the relationship between intervention coverage and effect sizes, and of the constraints on achieving high effective coverage, exposes flaws in the arguments about mathematical sensitivity to parameters that have been used to shift the focus away from certain interventions.^19^ Reaching a policy objective in situations with either high baseline or insecticide resistant vectors may be impossible using a single mode of vector control, so VC would need to be reduced using other methods. Species that are refractory to one intervention (e.g., IRS), may be sensitive to another (e.g., LSM), or a combination of interventions may enhance their effectiveness (Figure 3A). The sharpest reductions may come from targeting the dominant vector, but achieving some policy objectives (including malaria elimination) might require integrated vector management, which could involve attacking various vector species in different ways, or achieving very high effective coverage levels with multiple interventions (Figure 3B). Similarly, the development of novel vector control interventions, such as release of genetically modified mosquitoes,^73^ would also benefit from considering effective coverage limitations in different settings. Identifying novel methods that act additively or synergistically with the existing package of interventions may mean higher effect sizes can be reached.

The ecology and behavior of the vectors that transmit malaria parasites are so varied that a single approach cannot be universally applicable, especially when pursuing elimination.^39^ Stratifying transmission for control and tailoring interventions requires gathering information at the human and mosquito population level in addition to medical intelligence and identifying a combination of interventions that would reduce transmission most effectively to achieve policy objectives. Of particular use are range maps of vector species,^45,74^ vector bionomics, transmission intensity,^58^ and the frequency of insecticide resistance genes combined with an understanding of their functional significance. While not explored here, the role of baseline human immunity is also an important consideration, especially if vaccines are to be considered in a package of future interventions, or the same framework is to be expanded to diseases with sterilizing immunity.^61,75^ Mathematical models and medical intelligence can be used to set rational expectations, and troubleshooting methods should be developed to monitor and evaluate vector control when those expectations are not met. Surveillance data should be validated as an accurate measure of trends in pathogen transmission and disease, and assembled in a systematic way to iteratively update maps and inform vector control. Such systems would help policy makers spend their limited vector control funding more effectively, to layer on additional vector control methods when needed, and to reach policy endpoints.

### Conclusions

Over the past 15 years huge strides have been made in reducing malaria transmission by scaling-up coverage of interventions that have strong experimental and theoretical support. The change in goal to malaria elimination brings with it a new set of challenges. Past experiences suggest that the path to elimination is long, highly non-linear, costly and above all one that needs to be well planned and frequently re-evaluated.^55,71^ Combining detailed baseline assessments of transmission, vector population attributes and program operational constraints using models that have a sound theoretical understanding of elimination dynamics is essential for providing elimination policy makers with contemporary advice.

The general consideration of Macdonald's analysis points to the need for a better understanding of how control goals can be reached in different contexts. The new frontier for modeling mosquito-borne pathogen transmission is to understand the interplay between mosquito ecology and behavior, variable baselines including some areas with very high transmission, the operational constraints on control programs, and the best way to achieve often challenging policy objectives across different real world, dynamic contexts.
